# Role of maternal age and pregnancy history in risk of miscarriage: prospective register based study

**DOI:** 10.1136/bmj.l869

**Published:** 2019-03-20

**Authors:** Allen J Wilcox, Nils-Halvdan Morken, Clarice R Weinberg, Siri E Håberg, Maria C Magnus

**Affiliations:** 1 Centre for Fertility and Health, Norwegian Institute of Public Health, PO Box 222 Skøyen, N-0213 Oslo, Norway; 2 MRC Integrative Epidemiology Unit at the University of Bristol, Bristol, UK; 3 Department of Population Health Sciences, Bristol Medical School, Bristol, UK; 4 Epidemiology Branch, National Institute of Environmental Health Sciences, Durham, NC, USA; 5 Department of Clinical Science, University of Bergen, Bergen, Norway; 6 Department of Obstetrics and Gynecology, Haukeland University Hospital, Bergen, Norway; 7 Biostatistics and Computational Biology Branch, National Institute of Environmental Health Sciences, Durham, NC, USA

## Abstract

**Objectives:**

To estimate the burden of miscarriage in the Norwegian population and to evaluate the associations with maternal age and pregnancy history.

**Design:**

Prospective register based study.

**Setting:**

Medical Birth Register of Norway, the Norwegian Patient Register, and the induced abortion register.

**Participants:**

All Norwegian women that were pregnant between 2009-13.

**Main outcome measure:**

Risk of miscarriage according to the woman’s age and pregnancy history estimated by logistic regression.

**Results:**

There were 421 201 pregnancies during the study period. The risk of miscarriage was lowest in women aged 25-29 (10%), and rose rapidly after age 30, reaching 53% in women aged 45 and over. There was a strong recurrence risk of miscarriage, with age adjusted odds ratios of 1.54 (95% confidence interval 1.48 to 1.60) after one miscarriage, 2.21 (2.03 to 2.41) after two, and 3.97 (3.29 to 4.78) after three consecutive miscarriages. The risk of miscarriage was modestly increased if the previous birth ended in a preterm delivery (adjusted odds ratio 1.22, 95% confidence interval 1.12 to 1.29), stillbirth (1.30, 1.11 to 1.53), caesarean section (1.16, 1.12 to 1.21), or if the woman had gestational diabetes in the previous pregnancy (1.19, 1.05 to 1.36). The risk of miscarriage was slightly higher in women who themselves had been small for gestational age (1.08, 1.04 to 1.13).

**Conclusions:**

The risk of miscarriage varies greatly with maternal age, shows a strong pattern of recurrence, and is also increased after some adverse pregnancy outcomes. Miscarriage and other pregnancy complications might share underlying causes, which could be biological conditions or unmeasured common risk factors.

## Introduction

Miscarriage is a common outcome of pregnancy, with most studies reporting 12% to 15% loss among recognised pregnancies by 20 weeks of gestation.^1^ ^2^ ^3^ ^4^ Quantifying the full burden of miscarriage is challenging because rates of pregnancy loss are high around the time that pregnancies are clinically recognised. As a result, the total rate of recognised loss is sensitive to how early women recognise their pregnancies. There are also differences across countries and studies in distinguishing between miscarriage and stillbirth. Furthermore, the observed miscarriage rate is affected by the competing risk of induced abortion. A general lack of data on induced abortions has made it difficult to determine how seriously this competing risk distorts the estimation of miscarriage rates. Based on national registries or population based cohort studies, the reported risk of miscarriage in Sweden, Finland, and Denmark was between 12.9% and 13.5%.^5^ ^6^ ^7^ ****A previous Norwegian study included all women treated at one of the main hospitals in Oslo between 2000 and 2002, and estimated a miscarriage rate of 12% when taking into account induced abortions.^8^

Although the cause of most miscarriages is unknown, they presumably result from a complex interplay between parental age, genetic, hormonal, immunological, and environmental factors.^9^ ^10^ Genetic factors, including parental chromosomal rearrangements and abnormal embryonic genotypes or karyotypes, could underlie more than half of recurrent miscarriages.^10^ Maternal age is the strongest known risk factor. The risk of miscarriage is slightly elevated in the youngest mothers and then rises sharply in older mothers.^7^ ^11^ There could be shared underlying risk factors for miscarriage and other adverse pregnancy outcomes. Several studies have looked at the association between the history of miscarriages and the future risk of other pregnancy complications,^12^ ^13^ ^14^ ^15^ ^16^ ^17^ but less is known about how complications might predict the future risk of miscarriage.^18^ ^19^ ^20^

Pregnant women in Norway have access to free healthcare, and most women with recognised pregnancies who experience loss, or impending loss, contact healthcare services. There is a separate register for the mandatory registration of induced abortions. Thus, nearly all recognised pregnancies are registered in at least one of the national health registers. The high awareness and education among women regarding the early signs of pregnancy, combined with freely available healthcare and mandatory registration systems, make Norway a favourable setting for studying the risk of miscarriage.

The aim of the current study was to estimate the rate of miscarriage among Norwegian women and to evaluate the association with age and pregnancy history.

## Methods

The study population consisted of all registered pregnancies in Norway between 2009 and 2013, excluding ectopic pregnancies. Information on pregnancies came from three national health registries: the Medical Birth Register of Norway (established in 1967), the induced abortion register (established in 1979), and the Norwegian Patient Register (established in 2008). The birth register includes information on all deliveries and fetal losses after 12 gestational weeks. The mandatory abortion register contains anonymous information from all healthcare providers that perform induced abortions. We used this register to obtain information about the frequency of induced abortions according to maternal age and gestational week of the procedure. The patient register provides information on all women in contact with specialist healthcare services during pregnancy, which provides the opportunity to identify losses before 12 weeks that are not captured in the birth register. We linked information on live births and fetal deaths identified through the birth register and the patient register by using unique personal identification numbers.

### Pregnancy outcomes and identification of unique pregnancies

We identified live births and fetal deaths after 12 gestational weeks from the birth register. In the birth register, a fetal death at 20 gestational weeks or later, or with a birthweight of 500 g or more, was considered a stillbirth. Fetal deaths before 20 gestational weeks with a birthweight of less than 500 g were considered a miscarriage. To the extent possible, we have identified miscarriage as losses between 6 and 20 weeks, with exceptions as noted in this paragraph. The patient register did not record the gestational age at the time of the miscarriage, but we assume that all miscarriages identified in the patient register occurred before 12 completed gestational weeks, as they would otherwise have been recorded in the birth register. Hospital discharges in the patient register are coded according to ICD-10 (international classification of diseases, 10th revision). We included the following ICD-10 codes to capture early miscarriages: hydatidiform mole (O01); blighted ovum and non-hydatidiform mole (O02.0); missed abortion (O02.1); other specified abnormal products of conception (O02.8); abnormal product of conception, unspecified (O02.9); spontaneous abortion (O03); and threatened abortion (O02.0). Pregnancies in the patient register are not registered with unique pregnancy identification numbers, and follow-up visits during the same pregnancy could produce multiple registrations. Therefore, we took steps to ensure that records reflected unique pregnancies. Firstly, we required a minimum of 6 weeks (42 days) between two successive records of miscarriage to consider them distinct pregnancies. Secondly, we required that a record of a miscarriage in the patient register should be at least 6 weeks after a registered pregnancy to the same women in the birth register. Thirdly, we excluded registered miscarriages that occurred in the gestational period of a registered pregnancy to the same woman in the birth register. In the case of multiple fetuses, the outcome was regarded as a live birth if all deliveries resulted in live births, as a miscarriage if there was at least one miscarriage but no stillbirth, and as a stillbirth if at least one of the deliveries resulted in a stillbirth. A multiple birth could thus result in both a miscarriage and a stillbirth, but only if there was a discrepancy in birthweight between the fetuses.

### Pregnancy history

For the analysis of previous pregnancy outcomes, women were categorized as having no previous pregnancy, live birth, stillbirth, miscarriage, or neonatal death. Neonatal death was defined as death in the first 28 days after delivery. Women with previous deliveries outside of Norway have missing records for these pregnancies in the birth register (3%). The registered parity reveals if a woman has any missing birth records. Information on previous pregnancies was most likely to be missing if the current pregnancy ended in live birth (eTable 1). We also obtained information on complications in the previous live birth pregnancy, including preterm delivery (<37 gestational weeks), post-term delivery (≥42 gestational weeks), small for gestational age, large for gestational age, pre-eclampsia, gestational diabetes, and delivery by caesarean section. Small for gestational age was defined as a birthweight below the 10th centile, and large for gestational age as a birthweight above the 90th centile, according to sex and gestational week specific birthweight distributions. Delivery by caesarean section was further subdivided into emergency (acute), planned (elective), or unspecified.

For women born in Norway (75% of our population), we also obtained information from the birth register on the conditions of the woman’s own birth. These included whether the woman herself was delivered preterm, small for gestational age, large for gestational age, or in a pregnancy with pre-eclampsia.

### Statistical analysis

We calculated the rate of miscarriages as the number of miscarriages in all ongoing pregnancies in each gestational week. An induced abortion that occurs at a gestational age when most miscarriages would have already occurred should be in the denominator at least as a partial count, or otherwise the apparent rate of miscarriage will be inflated. In the absence of data on the gestational week of induced abortion (which is rarely available), there is no precise way to take the induced abortions into account. One practical approach that has been suggested is to include half of the induced abortions in the denominator.^21^

The Norwegian data provide distributions by gestational week for all induced abortions according to maternal age, which allowed us to directly estimate the number of pregnancies that would have resulted in a miscarriage if the pregnancy had not been terminated. Because the Norwegian data do not include information on gestational age for miscarriages, we applied rates of gestational week specific risk of miscarriage derived from a population with miscarriage rates similar to those in Norway.^22^ We subsequently estimated the risk of miscarriage by adding to the numerator the estimated number of induced abortions that would have ended in a miscarriage, and including in the denominator all pregnancies ending in induced abortions (see the online supplement for details).

The majority of induced abortions in Norway occurred soon after recognition of pregnancy, so that our correction for induced abortions made only a very small difference in the estimated overall risk of miscarriages. This was also true when we calculated the age associated risk of miscarriage, for which there were large differences in the occurrence of induced abortions by maternal age. With this reassurance that the early induced abortions in Norway produce little distortion in the risk of miscarriage, we excluded induced abortions from the remaining analyses.

We calculated the odds ratio of miscarriage according to pregnancy history by logistic regression, using cluster variance estimation to account for women with multiple pregnancies during the study period. To evaluate the recurrence risk of miscarriage, we examined the risk among women who had one, two, and three previous consecutive miscarriages, as compared with the risk of miscarriage in women having their first pregnancy. Given the strong and non-linear influence of maternal age on the risk of miscarriage, the multivariable model adjusted for both age and age squared.^7^ We conducted a sensitivity analysis of the risk according to the outcome of the previous pregnancy adjusting for interpregnancy interval, where we assigned all miscarriages to have a gestational age of 8 weeks. We also conducted a sensitivity analysis of the risk according to complications in the previous pregnancy where we adjusted for maternal smoking during the previous pregnancy, for the approximately 80% of pregnancies with this information available. All analyses were conducted by using Stata version 14 (Statacorp, College Station, TX).

### Patient and public involvement

No patients were involved in setting the research question or the outcome measures, nor were they involved in developing plans for recruitment, design, or implementation of the study. No patients were asked to advise on interpretation or writing up of results. There are no plans to disseminate the results of the research to study participants or the relevant patient community.

## Results

**Fig 1:**
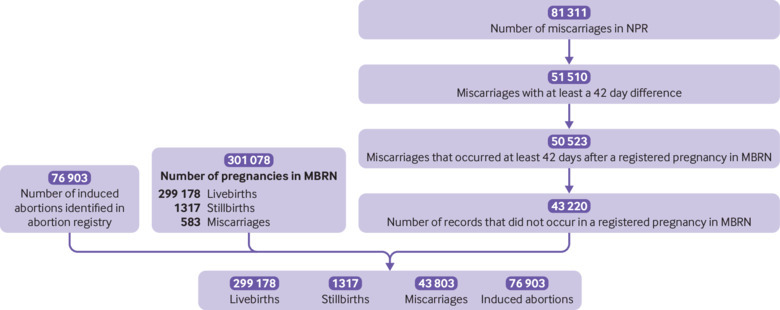
Pregnancies identified in the Medical Birth Register of Norway (MBRN), the induced abortion register, and the Norwegian Patient Register (NPR) between January 2009 and December 2013

Figure 1 shows that there were 421 201 pregnancies registered in Norway between 2009 and 2013. Among these, 299 178 resulted in live birth, 1317 in stillbirth, 43 803 in miscarriage, and 76 903 in induced abortion. After correcting for induced abortions, the overall risk of miscarriage was 12.8%. We show the proportion of miscarriages at <12 gestational weeks, <14 gestational weeks, <20 gestational weeks, <22 gestational weeks, and <24 gestational weeks in eTable 2.

### Age specific risk of miscarriage

**Fig 2:**
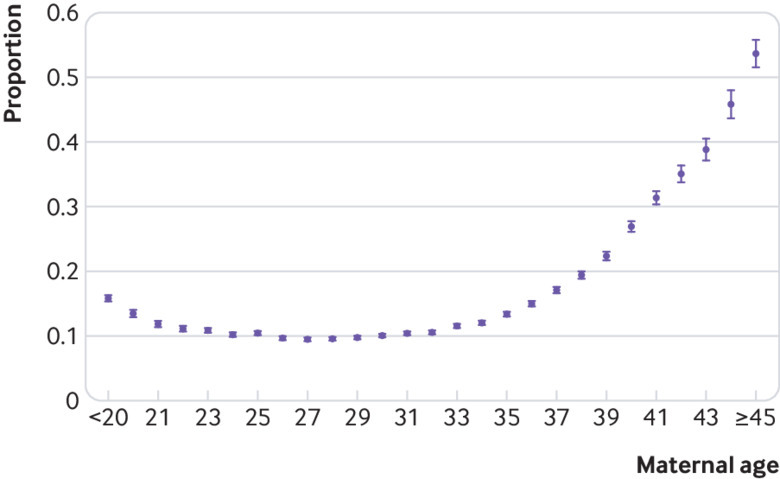
Risk of miscarriage according to maternal age. The bars for each point reflect the 95% confidence intervals

Table 1 shows that the risk of miscarriage varied substantially across age groups. Figure 2 shows that the age associated risk of miscarriage has a J shaped pattern. The risk of miscarriage was lowest among women aged 25-29 (9.8%), with the absolute lowest risk at age 27 (9.5%), and the highest risk at age 45 and over (53.6%). The youngest mothers (<20 years) had a risk of 15.8%. Table 1 shows that adjusting for induced abortions modestly decreased the risk of miscarriage for the oldest and youngest women, with scarcely any change in the total risk.

**Table 1:** Frequency of pregnancy outcomes in Norway between 2009 and 2013 by maternal age. Values are numbers (percentages) unless stated otherwise

Age (years)	Pregnancies	Live births	Stillbirths	Induced abortions		Miscarriage	
						Excluding induced abortions*	Including induced abortions†
<20	17 066	5792 (33.9)	28 (0.2)	10 083 (59.1)		1163 (16.7)	2698 (15.8)
20-24	70 829	43 373 (61.2)	172 (0.2)	21 811 (30.8)		5473 (11.2)	7987 (11.3)
25-29	122 137	94 022 (77.0)	352 (0.3)	17 591 (14.4)		10 172 (9.7)	11 910 (9.8)
30-34	123 266	97 631 (79.2)	402 (0.3)	13 312 (10.8)		11 921 (10.8)	13 362 (10.8)
35-39	68 502	48 590 (70.9)	300 (0.4)	9793 (14.3)		9819 (16.7)	11 414 (16.7)
40-44	18 076	9340 (51.7)	59 (0.3)	4004 (22.2)		4673 (33.2)	5820 (32.2)
>45	1315	430 (32.7)	4 (0.3)	309 (23.5)		572 (56.9)	705 (53.6)
Missing information	10	0	0	0		10 (NA)	0
Total	421 201	299 178 (71.0)	1317 (0.3)	76 903 (18.3)		43 803 (12.7)	53 906 (12.8)

* From the denominator.

† That would have resulted in a miscarriage in the numerator and all induced abortions in the denominator.

### Pregnancy history and risk of miscarriage

Table 2 shows that the risk of miscarriage was increased in women whose previous pregnancy ended in a stillbirth (adjusted odds ratio 1.30, 95% confidence interval 1.11 to 1.53) or miscarriage (1.65, 1.59 to 1.71), compared with women with no previous pregnancy. The risk was also higher for women with a history of neonatal death, although the numbers were small and the estimate was imprecise. Associations were similar in our sensitivity analysis adjusting for interpregnancy interval (eTable 3). There was a strong recurrence risk of miscarriage, which remained after adjustment for maternal age. Table 3 shows that after one miscarriage, the adjusted odds ratio of another was 1.54 (95% confidence interval 1.48 to 1.60). This increased to 2.21 (2.03 to 2.41) after two consecutive miscarriages, and 3.97 (3.29 to 4.78) after three or more consecutive miscarriages.

**Table 2:** Risk of miscarriage in Norway between 2009 and 2013 according to the outcome of the previous pregnancy (n=315 963*)

Previous pregnancy	Total number of pregnancies excluding induced abortions	Number of miscarriages (%)	Adjusted† odds ratio (95% CI)
No previous pregnancy	127 150	14 791 (11.6)	Ref
Live birth	157 763	19 170 (12.2)	0.91 (0.89 to 0.94)
Stillbirth	1175	205 (17.5)	1.30 (1.11 to 1.53)
Miscarriage	29 434	6214 (21.1)	1.65 (1.59 to 1.71)
Neonatal death	441	75 (17.0)	1.28 (0.97 to 1.64)

* 105 238 pregnancies excluded from the analysis because of unknown pregnancy history or because the previous or current pregnancy was an induced abortion.

† Adjusted for age.

**Table 3:** Recurrence risk of miscarriage in Norway between 2009 and 2013 after consecutive miscarriages (n=156 584*)

Previous miscarriages	Total number of pregnancies excluding induced abortions	Number of miscarriages (%)	Adjusted† odds ratio (95% CI)
No previous pregnancy	127 150	14 791 (11.6)	Ref
1	25 575	5051 (19.8)	1.54 (1.48 to 1.60)
2	3208	890 (27.7)	2.21 (2.03 to 2.41)
3 or more	651	273 (41.9)	3.97 (3.29 to 4.78)

* 264 617 pregnancies excluded from the analysis because of unknown pregnancy history or because the previous or current pregnancy was an induced abortion or because the previous pregnancy was a live birth or a stillbirth.

† Adjusted for age.

### Pregnancy complications and risk of miscarriage

Other adverse outcomes in previous pregnancies also predicted a higher risk of miscarriage. Table 4 shows that the risk of miscarriage was higher if the previous live birth was preterm (adjusted odds ratio 1.22, 95% confidence interval 1.12 to 1.29), if the previous pregnancy included a diagnosis of gestational diabetes (1.19, 1.05 to 1.36), or if the delivery method was caesarean section (1.16, 1.12 to 1.21). Women whose previous delivery had been post-term had a slightly reduced risk of miscarriage (0.84, 0.79 to 0.90). There was weak evidence for an increased risk after delivery of infants small for gestational age, large for gestational age, or with a congenital malformation. Pre-eclampsia in the previous pregnancy was not associated with increased risk of miscarriage. The associations between complications in the previous pregnancy and miscarriage remained similar when adjusting for the interpregnancy interval and smoking in the previous pregnancy (eTable 4).

**Table 4:** Risk of miscarriage in Norway between 2009 and 2013 according to complications in the previous live birth pregnancy (n=158 204*)

Exposure	Total number of pregnancies excluding induced abortions	Number of miscarriages (%)	Adjusted† odds ratio (95% CI)
Gestational age at delivery:			
Preterm	8639	1261 (14.7)	1.22 (1.12 to 1.29)
Term	136 286	16 436 (12.2)	Ref
Post-term	11 602	1272 (11.1)	0.84 (0.79 to 0.90)
Fetal growth:			
Small for gestational age	13 014	1642 (12.6)	1.06 (1.01 to 1.12)
Normal for gestational age	130 366	15 595 (12.0)	Ref
Large for gestational age	13 109	1727 (13.2)	1.05 (0.99 to 1.10)
Congenital malformation:			
No	150 791	18 288 (12.1)	Ref
Yes	7413	957 (12.9)	1.07 (0.99 to 1.14)
Pre-eclampsia:			
No	152 266	18 505 (12.2)	Ref
Yes	5938	740 (12.5)	1.04 (0.96 to 1.13)
Gestational diabetes:			
No	156 405	18 962 (12.1)	Ref
Yes	1799	283 (15.7)	1.19 (1.05 to 1.36)
Caesarean section:			
No	135 858	16 029 (11.8)	Ref
Yes	22 346	3216 (14.4)	1.16 (1.12 to 1.21)
Acute	6284	1047 (16.7)	1.29 (1.20 to 1.39)
Elective	15 918	2149 (13.5)	1.11 (1.06 to 1.17)
Unspecified	144	20 (13.9)	1.04 (0.63 to 1.70)

* 127 150 pregnancies excluded because the woman had no previous registered pregnancy; 1175 pregnancies excluded because the previous pregnancy was a stillbirth, 29 434 pregnancies excluded because the previous pregnancy was a miscarriage; and 105 238 pregnancies excluded because the outcome of the previous pregnancy was unknown or an induced abortion.

† Adjusted for age.

The outcome of pregnancies of women for whom we had information from their own birth record was very similar to outcomes for women lacking this information (eTable 5). Table 5 shows that using information from the women’s own birth record, we identified a modest increased risk of miscarriage if the woman herself had been born small for gestational age (adjusted relative risk 1.08, 95% confidence interval 1.04 to 1.13). In contrast, there was no evidence of increased risk for women exposed to pre-eclampsia in utero, or for women born large for gestational age, preterm, or post-term.

**Table 5:** Risk of miscarriage in Norway between 2009 and 2013 according to the women’s own birth record (n=258 954*)

Exposure	Total number of pregnancies excluding induced abortions	Number of miscarriages (%)	Adjusted† odds ratio (95% CI)
Gestational age at delivery:			
Preterm	10 599	1396 (13.2)	1.05 (0.99 to 1.12)
Term	194 910	24 462 (12.6)	Ref
Post-term	38 044	4955 (13.0)	1.04 (1.00 to 1.08)
Fetal growth:			
Small for gestational age	26 397	3598 (13.6)	1.08 (1.04 to 1.13)
Normal for gestational age	195 358	24 571 (12.6)	Ref
Large for gestational age	21 548	2618 (12.2)	0.97 (0.93 to 1.02)
Pre-eclampsia:			
No	252 346	31 951 (12.7)	Ref
Yes	6608	852 (12.9)	1.06 (0.98 to 1.15)

* 76 903 pregnancies excluded because they resulted in an induced abortion; 1412 pregnancies excluded because the woman was born before the medical birth register was set up (1967); and 83 932 pregnancies excluded because the woman was born outside of Norway.

† Adjusted for age.

## Discussion

Miscarriage is a common outcome of pregnancy, but the rate is challenging to estimate because of inconsistent registration and documentation. Few countries have population registries that include miscarriage. In Norway, miscarriage data has been consistently collected since 2008. In this first description of the Norwegian register data, we can confirm some observations with new precision, by using contemporary, comprehensive national data from a high income country.

### Strengths and weaknesses of this study

Important strengths of our study include the population based design, the prospective collection of the data, and the availability of information from the woman’s own birth record. Limitations include likely under ascertainment of early miscarriages. The patient register captures only miscarriages that led to a consultation with specialist healthcare services. Women who had contact only with their general practitioner are therefore not in the patient register. However, in Norway, most women who recognise a miscarriage are likely to receive care from a specialist as these will be able to provide ultrasound confirmation on the pregnancy status. All prenatal care in Norway is free of charge and available to all women in the country. Births to Norwegians outside Norway are not registered in the birth register. For some women we therefore lacked information on the outcome of the woman’s previous pregnancy. Also, for women born outside Norway, her own birth record was not in the register, and we did not know whether she herself was preterm, small for gestational age, etc. However, there was little difference in pregnancy outcomes in women with and without this information.

The associations of risk of miscarriage with complications in previous pregnancies point to the presence of causal factors that increase the risk of both. Information on potential causal factors is limited in the national registries. We can show that maternal smoking does not contribute to the associations, but we lack information on paternal age, maternal ethnicity, education, and body mass index. Ethnicity could be important since we know that 14% of Norwegians are immigrants (and ~25% of pregnancies in the current study were to women born outside of Norway).^23^ Although most immigrants are from other Nordic and eastern European countries, there are immigrant groups that possibly have a greater underlying risk of miscarriage compared with ethnic Norwegians.^24^ ^25^ ^26^ More focused studies with more detailed information might be able to identify new underlying causes for both miscarriage and related pregnancy complications.

The overall risk of miscarriage among recognised pregnancies in Norway was 12.8%. This risk is remarkably similar to reports from other Nordic countries (range of 13% to 14%).^5^ ^6^ ^7^ Estimates from the United States and Canada were more variable (range of 9% to 20%).^2^ ^3^ ^4^ This consistency with other Nordic studies, and with prospective studies with full ascertainment of early miscarriages, provides some reassurance that the Norwegian registries capture most recognised miscarriages. Future research could estimate the extent of Norwegian miscarriages managed in a non-specialist setting (eg, by general practitioners, community nurses, or midwives).

As expected,^7^ ^11^ the risk of miscarriage was strongly related to maternal age. The risk was moderately increased (15.8%) for women under the age of 20, with the absolute lowest risk (9.5%) at age 27, and then rising nearly linearly after the age of 30 to reach 54% at ages 45 and over (fig 2). The increased risk among young women is a curious finding. In a Danish study, the apparent increase among younger women did not persist after a crude adjustment for induced abortions.^7^ When we made a similar crude adjustment (adding half of induced abortions to the denominator), the increase among the youngest women was likewise mostly removed, but when we made the more precise adjustment, a higher risk among the youngest women persisted. This could indicate unrecognised social causes of miscarriage, or an effect of reproductive immaturity. The fact that our careful adjustment for induced abortions made little difference to any of the age estimates is presumably owing to the fact that induced abortions in Norway occur too early (84% in the first 9 weeks) to materially interfere with the miscarriage calculation.

Women vary in their risk of miscarriage at a given age, for reasons that are not well understood. A miscarriage marks a woman as being at relatively higher risk, and this risk is expressed in subsequent pregnancies. Controlling for maternal age, the odds ratio for miscarriage increased from 1.5 after one miscarriage to 2.2 after two and 4.0 after three. Recurrence risk has been previously reported, although not with this precision or to this extent.^27^

We found evidence that certain other pregnancy outcomes cluster with the risk of miscarriage, suggesting that these outcomes might share underlying causes. Specifically, the risk of miscarriage was moderately increased among women who had experienced a stillbirth, preterm delivery, or gestational diabetes in their previous pregnancy. No previous studies have considered those pregnancy outcomes as risk factors for miscarriage. Our results for preterm delivery are supported by the temporally reverse association, with previous studies reporting a higher risk of preterm birth among women with a history of miscarriage.^14^ ^16^ ^28^ ^29^ ^30^ ^31^ Possible shared pathways include cervical insufficiency and infections,^14^ ^16^ ^28^ ^29^ ^30^ ^31^ although these are speculative. There are a few clues in the literature suggesting that glucose metabolism abnormalities could increase both the risk of miscarriage and preterm delivery.^32^ ^33^ ^34^ ^35^

Unexpectedly, there was a small increased risk of miscarriage among women who were small for gestational age at their own birth. There are no obvious mechanisms for this finding, and it remains to be confirmed in future studies. However, there could be shared genetic or risk related exposures between mothers and daughters (eg, smoking), that potentially explain this association.

We also observed a small increase in the risk of miscarriage after a caesarean section. A systematic review of caesarean section and subsequent risk of miscarriage has reported inconsistent effects, with relative risk or odds ratio estimates ranging from 0.76 to 1.32.^19^ This systematic review did not pool the results from individual studies owing to the heterogeneity among studies, including varying definitions of miscarriage or early fetal demise. It is possible that the underlying problem leading to delivery by caesarean section also increases the risk of miscarriage in the subsequent pregnancy.^19^ We did not have sufficient detailed information to explore the role of factors necessitating delivery by caesarean section.

### Conclusion

Population based data from Norway provide precise estimates of the risk of miscarriage related to maternal age, with the lowest risk at age 27. The risk of miscarriage increases as much as fourfold after three consecutive previous miscarriages, implying considerable variability in risk between couples. Exploratory associations suggest that the risk of miscarriage is linked to some previous pregnancy complications (stillbirth, preterm delivery, and gestational diabetes). More focused studies of these associations might lead to new insights regarding the shared underlying causes of pregnancy complications and miscarriage.

**What is already known on this topic**

Miscarriage is a common pregnancy outcome with a substantial recurrence risk
The risk of miscarriage increases strongly with maternal age
There is a small increase in the risk of miscarriage in the youngest mothers

**What this study adds**

The modest increased risk of miscarriage among the youngest mothers (<20 years) persisted after accounting for induced abortions
The risk of miscarriage was increased if the previous pregnancy ended in a preterm delivery, caesarean section, or if the woman had gestational diabetes
Women who themselves were born small for gestational age had an increased risk of miscarriage

## Web Extra

Extra material supplied by the author

Supplementary information: Online supplement methods

Supplementary information: eTable 1-5

## Notes

Contributors: MCM, AJW, and SEH conceived and designed the study. SEH obtained access to data. MCM conducted the data analysis and drafted the initial version of the manuscript. CRW and NHM provided important insight during the data analysis. All authors contributed in the interpretation of the data and critically revised the manuscript. All authors had full access to the data in the study and can take responsibility for the integrity of the data and the accuracy of the data analysis. MCM is the guarantor. The corresponding author attests that all listed authors meet authorship criteria and that no others meeting the criteria have been omitted.

Ethical approval: The national register linkage was approved by the Regional Committee for Medical and Health Research Ethics for South/East Norway (2014/404).

Data sharing: No additional data are available.

The lead author (MCM) affirms that the manuscript is an honest, accurate, and transparent account of the study being reported; that no important aspects of the study have been omitted; and that any discrepancies from the study as planned have been explained.
