# Supplementary material for: Role of maternal age and pregnancy history in risk of miscarriage: prospective register based study
Source: BMJ. 2019 Mar 20;364:l869. doi: 10.1136/bmj.l869 (PMC6425455; doi:10.1136/bmj.l869)
Supplement: Supplementary file 1 — Supplementary information: Online supplement methods [file magm048089.ww1.pdf]

Online supplement methods

We estimated the number of induced abortions that would have resulted in a miscarriage if the pregnancy had not been terminated, using published data on the gestational-week-specific risk of miscarriage.[19] We used this published data to estimate the cumulative probability of fetal death up to and including gestational week 20 for pregnancies terminated at different gestational weeks. The frequency of induced abortions according to maternal age gestational week at the time of the procedure was available from the abortion registry.

The expected number of miscarriage per induced abortion that happens at week k will be computable as 1 minus the probability that a k-week pregnancy survives to week 20:

Let

$$R_k = exp\left\{\sum_{j=k}^{20} \ln(1 - p_j)\right\}$$

This denotes the probability of survival to week 20, given alive at the start of week k (when the induced abortion occurred), so that 1 minus that is the probability of miscarriage given survival up to week k.

After having randomly assigned a proportion of the induced abortions to have resulted in a miscarriage based on this calculated cumulative probability of miscarriage, we estimated the age specific relative risks (RR) of miscarriage using age 26 as the reference, as this was the median age of women in the publication from which we obtained the gestational week specific risk of miscarriage. We then used these initial estimated age-specific RRs to calculate maternal age and gestational week specific cumulative risk of miscarriage, and used these estimated probabilities to randomly assign a proportion of induced abortions to have resulted in a miscarriage. This process was repeated for a total of 40 iterations until we saw that the age-specific RRs of miscarriage were approaching their limit values. To estimate the confidence intervals of the age-specific risk of miscarriage, we then used the final obtained age-specific RRs to randomly assign the proportion of induced abortions that would have resulted in a miscarriage for a total of 1000 imputed data sets. The absolute proportion of miscarriage was then estimated as the mean across these imputed datasets. The confidence intervals for the proportions were then estimated using Rubin's rules:

$$\sigma^2 = \bar{U} + \left\{1 + \frac{1}{m}\right\} B$$

Where

$$\bar{U} = \frac{1}{m}\{\hat{p}_j(1 - \hat{p}_j)/n_j\}$$

is the within imputation variance;

And

$$B = \frac{1}{m-1}\left\{\sum_{j=k}^{m} (\hat{p}_j - \bar{p})\right\}\text{^}2$$

is the between imputation variance.
