# Supplementary material for: Role of maternal age and pregnancy history in risk of miscarriage: prospective register based study
Source: BMJ. 2019 Mar 20;364:l869. doi: 10.1136/bmj.l869 (PMC6425455; doi:10.1136/bmj.l869)
Supplement: Supplementary file 2 — Supplementary information: eTable 1-5 [file magm048089.ww2.pdf]

eTable 1 Age and pregnancy outcome among women with and without information on the outcome of their previous pregnancy

	Included in the analysis of prior pregnancy outcome (N=315,963)	Excluded from analysis because of missing information on prior pregnancy outcome* (N= 10,882)
Age at delivery, Mean (SD)	30.1 (5.4)	29.5 (5.7)
Pregnancy outcome, % (N)		
Live birth	86.8 (274,298)	87.8 (24,880)
Stillbirth	0.4 (1,210)	0.4 (107)
Miscarriage	12.8 (40,455)	11.8 (3,348)

These women had birth records which indicated that they had experienced a prior pregnancy, or had two subsequent birth records which indicated that they had had a pregnancy between the two recorded pregnancies, but for whom we did not know the outcome of the previous pregnancy.

eTable 2 Frequency of miscarriage according to different gestational week cut-offs

N=421,201

**Gestational week cut-off**	**Proportion miscarriages adjusted for induced abortions<br>% (N)**
<12 gestational weeks	11.9 (50,154)
<14 gestational weeks	12.2 (51,447)
<20 gestational weeks	12.8 (53,906)
<22 gestational weeks	12.9 (54,402)
<24 gestational weeks	12.9 (54,464)

eTable 3 Risk of miscarriage according to whether **the previous pregnancy** ended in a live birth, stillbirth, miscarriage or a neonatal death (n= 315,963*)

**Previous pregnancy**	**Total number of pregnancies excluding induced abortions**	**Number of miscarriages % (N)[a]**	**Adjusted for age OR (95% CI)**	**Adjusted for age and inter-pregnancy inteval**
Live birth	157,763	12.2 (19,170)	Ref	Ref
Stillbirth	1,175	17.5 (205)	1.43 (1.22 to 1.67)	1.57 (1.34 to 1.84)
Miscarriage	29,434	21.1 (6,214)	1.80 (1.74 to 1.86)	2.03 (1.93 to 2.13)
Neonatal death	441	17.0 (75)	1.38 (1.07 to 1.79)	1.52 (1.17 to 1.19)

*105,238 pregnancies excluded from the analysis because of unknown pregnancy history or because their previous or current pregnancy was an induced abortion.

eTable 4 Risk of miscarriages by whether **the previous pregnancy** was a live birth with a pregnancy complication (n= 158,204)

**Exposure**	**Exposure group**	**Total number of pregnancies excluding induced abortions**	**Number of miscarriages % (N)**	**Adjusted for age OR (95% CI)**	**Adjusted for age and inter-pregnancy interval OR (95% CI)**	**Adjusted for age, inter-pregnancy interval and smoking OR (95% CI)***
Gestational age	Preterm delivery	8,639	14.7 (1,261)	1.22 (1.12 to 1.29)	1.20 (1.13 to 1.28)	1.19 (1.11 to 1.28)
	Term delivery	136,286	12.2 (16,436)	Ref	Ref	Ref
	Post-term delivery	11,602	11.1 (1,272)	0.84 (0.79 to 0.90)	0.84 (0.79 to 0.90)	0.84 (0.79 to 0.91)
Fetal growth	Small-for-gestational age	13,014	12.6 (1,642)	1.06 (1.01 to 1.12)	1.06 (1.00 to 1.12)	1.04 (0.97 to 1.10)
	Normal-for-gestational-age	130,366	12.0 (15,595)	Ref	Ref	Ref
	Large-for-gestational-age	13,109	13.2 (1,727)	1.05 (0.99 to 1.10)	1.03 (0.97 to 1.09)	1.03 (0.97 to 1.10)
Congenital malformation	No	150,791	12.1 (18,288)	Ref	Ref	Ref
	Yes	7,413	12.9 (957)	1.07 (0.99 to 1.14)	1.07 (1.00 to 1.15)	1.07 (0.99 to 1.16)
Preeclampsia	No	152,266	12.2 (18,505)	Ref	Ref	Ref
	Yes	5,938	12.5 (740)	1.04 (0.96 to 1.13)	1.03 (0.95 to 1.11)	1.02 (0.93 to 1.12)
Gestational diabetes	No	156,405	12.1 (18,962)	Ref	Ref	Ref
	Yes	1,799	15.7(283)	1.19 (1.05 to 1.36)	1.25 (1.10 to 1.43)	1.22 (1.06 to 1.41)
Caesarean section	No	135,858	11.8 (16,029)	Ref	Ref	Ref
	Yes	22,346	14.4 (3,216)	1.16 (1.12 to 1.21)	1.18 (1.13 to 1.23)	1.17 (1.12 to 1.23)
	Acute	6,284	16.7 (1,047)	1.29 (1.20 to 1.39)	1.31 (1.22 to 1.41)	1.32 (1.21 to 1.43)
	Elective	15,918	13.5 (2,149)	1.11 (1.06 to 1.17)	1.13 (1.08 to 1.19)	1.12 (1.06 to 1.18)
	Unspecified	144	13.9 (20)	1.04 (0.63 to 1.70)	0.97 (0.59 to 1.60)	1.11 (0.64 to 1.91)

*Restricted to the 80% of pregnancies that had information on smoking in the previous pregnancy. This models adjusts for maternal smoking in the prior pregnancy.

Small-for-gestational-age was defined as birthweight below the 10th percentile, and large-for-gestational-age as birthweight above the 90th percentile, using national sex-and-gestational-week-specific birthweight distributions (i.e. the distribution among all deliveries in Norway as registered in the Medical Birth Register).

Preterm delivery was defined as <37 gestational weeks and post-term delivery was defined as ≥42 gestational weeks.

eTable 5 Age and pregnancy outcome among women with and without information from their own birth record

	Information from the woman's own birth record (N=258,954)	No information from the woman's own birth record (N=85,344)
Age at delivery, Mean (SD)	29.9 (5.4)	30.5 (5.6)
Pregnancy outcome, % (N)		
Live birth	87.0 (225,226)	87.0 (73,952)
Stillbirth	0.4 (925)	0.5 (392)
Miscarriage	12.7 (32,803)	12.9 (11,000)
